# Persistent microbial communities in hyperarid subsurface habitats of the Atacama Desert: Insights from intracellular DNA analysis

**DOI:** 10.1093/pnasnexus/pgae123

**Published:** 2024-04-23

**Authors:** Lucas Horstmann, Daniel Lipus, Alexander Bartholomäus, Felix Arens, Alessandro Airo, Lars Ganzert, Pedro Zamorano, Dirk Schulze-Makuch, Dirk Wagner

**Affiliations:** GFZ German Research Centre for Geosciences, Section Geomicrobiology, 14473 Potsdam, Germany; Department Experimental Phycology and Culture Collection of Algae (EPSAG), Albrecht-von-Haller-Institute for Plant Sciences, Georg August University Göttingen, 37073 Göttingen, Germany; GFZ German Research Centre for Geosciences, Section Geomicrobiology, 14473 Potsdam, Germany; GFZ German Research Centre for Geosciences, Section Geomicrobiology, 14473 Potsdam, Germany; Zentrum für Astronomie und Astrophysik, Technische Universität Berlin, 10623 Berlin, Germany; Museum für Naturkunde, Leibniz-Institut für Evolutions- und Biodiversitätsforschung, 10115 Berlin, Germany; Department of Plankton and Microbial Ecology, Leibniz Institute of Freshwater Ecology and Inland Fisheries, 16775 Stechlin, Germany; Laboratorio de Microorganismos Extremófilos, University of Antofagasta, Antofagasta 02800, Chile; GFZ German Research Centre for Geosciences, Section Geomicrobiology, 14473 Potsdam, Germany; Zentrum für Astronomie und Astrophysik, Technische Universität Berlin, 10623 Berlin, Germany; Department of Plankton and Microbial Ecology, Leibniz Institute of Freshwater Ecology and Inland Fisheries, 16775 Stechlin, Germany; GFZ German Research Centre for Geosciences, Section Geomicrobiology, 14473 Potsdam, Germany; Institute of Geosciences, University of Potsdam, 14476 Potsdam, Germany

**Keywords:** Atacama, hyperarid, desert soil, subsurface, microbial community

## Abstract

Desert environments constitute one of the largest and yet most fragile ecosystems on Earth. Under the absence of regular precipitation, microorganisms are the main ecological component mediating nutrient fluxes by using soil components, like minerals and salts, and atmospheric gases as a source for energy and water. While most of the previous studies on microbial ecology of desert environments have focused on surface environments, little is known about microbial life in deeper sediment layers. Our study is extending the limited knowledge about microbial communities within the deeper subsurface of the hyperarid core of the Atacama Desert. By employing intracellular DNA extraction and subsequent 16S rRNA sequencing of samples collected from a soil pit in the Yungay region of the Atacama Desert, we unveiled a potentially viable microbial subsurface community residing at depths down to 4.20 m. In the upper 80 cm of the playa sediments, microbial communities were dominated by Firmicutes taxa showing a depth-related decrease in biomass correlating with increasing amounts of soluble salts. High salt concentrations are possibly causing microbial colonization to cease in the lower part of the playa sediments between 80 and 200 cm depth. In the underlying alluvial fan deposits, microbial communities reemerge, possibly due to gypsum providing an alternative water source. The discovery of this deeper subsurface community is reshaping our understanding of desert soils, emphasizing the need to consider subsurface environments in future explorations of arid ecosystems.

**Significance Statement**

Microorganisms are the main actors by controlling nutrient fluxes in hyperarid desert ecosystems. However, only few studies have explored microbial communities of desert subsurface soils. Our study used intracellular DNA to characterize the living microbial community along a soil profile in the Atacama Desert and revealed diverse communities down to a depth of 4.20 m. This discovery is expanding our understanding of desert biodiversity suggesting that subsurface niches are colonized under favorable environmental conditions, implying that the global biodiversity of deserts might have been underestimated in previous studies. An example of favored conditions is the presence of a gypsum-containing geological substrate, providing water by dewatering to anhydrite and thus being a potential analog for subsurface habitable niches on Mars.

## Introduction

Despite being characterized by some of the harshest and life-threating conditions on Earth, extreme desert environments harbor microbial life and thus represent unique ecosystems exhibiting an exceptional resilience to endure. Microorganisms are the main and often only biological component in these environments and are driving major ecological functions including nutrient cycling, soil formation, and water preservation, often without major input of moisture by precipitation (1).

This applies especially to the 105,000 km^2^ large Atacama Desert in northern Chile, which is considered the driest hot desert in the world and therefore a highly relevant location to study life under extreme aridity. A comprehensive 4-year study conducted by McKay et al. (2) in the hyperarid region of the Yungay Valley revealed only one rain event of 2.3 mm during the entire observation period, underscoring the desert’s remarkably dry characteristics. Due to these harsh environmental conditions where chemical weathering, erosion, and biological activity are nearly absent, the processes of soil formation differ significantly from those observed in other regions on our planet, resulting in the accumulation of atmospheric particles including salts and dust (3, 4). This leads to the presence of various salts of sulfates and halides but also highly soluble salts of nitrate, iodate, and perchlorate (5).

With higher forms of life largely being absent, the hyperarid soils are mainly dominated by bacteria (6–8). These soils are inhabited by a diverse range of taxa from phyla such as Acidobacteriota, Proteobacteria, Chloroflexi, Firmicutes, and Actinobacteriota. Especially taxa affiliated to Actinobacteriota, particularly those associated with common soil and desert species/genera, can be detected (7, 9–11). Communities in samples from the atmosphere–soil interface comprise organisms with a high tolerance toward desiccation and UV radiation, e.g. *Rubrobacter* species (8).

Hypolithic and endolithic microorganisms have been found to inhabit various substrates in the Atacama Desert, such as halite (12, 13) and quartz crystals (13–15). These niches are primarily occupied by cyanobacteria, often in association with salt- and desiccation-tolerant Actinobacteriota and nitrogen-fixing Alphaproteobacteria (12, 13, 15). However, there are exceptions, such as so-called red hypoliths, which are dominated by taxa of the phylum Chloroflexi (15). These niches provide favorable conditions for microorganisms, including increased moisture levels due to the hygroscopic properties of salts (12, 16). Endolithic microbial communities have also been found to colonize gypsum crystals (13, 17). The porous matrix of the crystal structure forms a microclimate, which protects microbes from ultraviolet radiation while providing enough light for photosynthesis (17, 18).

Studying microbial diversity and distribution is essential to fully understand the pivotal role of microbial-driven processes in maintaining the ecological balance and functionality of desert ecosystems and its future development, particular against the backdrop of a changing climate. This is especially important when considering the largely unexplored microbial populations residing in deeper subsurface horizons (>100 cm depth). Shallow horizons (0–80 cm depth) of desert subsurface sediments have been suggested to represent niche environments shielded from UV radiation and with higher water availability (11). Therefore, these subsurface conditions may provide essential nutrients for microbial life and metabolic activity. Despite initial efforts using clone sequencing and a Life Detector Chip and recent next generation sequencing (NGS)-based efforts to assess the biodiversity of the deeper layers of the Atacama soils down to a depth of 520 and 340 cm (19, 20), knowledge about microbial distribution and microbial processes in deeper Atacama sediments is still limited, as most of the other available datasets primarily focus on the upper sediment layers down to approximately 100 cm in maximum (8, 11). Consequently, it is necessary to further evaluate microbial life in deeper sediments to obtain a better understanding if and how microorganisms exist and survive in these settings.

In recent years, cultivation-independent methods have significantly advanced our understanding of microbial diversity in environmental settings, surpassing the limitations of traditional cultivation-based approaches. NGS methods have played a pivotal role in this progress by generating vast amounts of microbial sequence data from a single sample, unraveling the community structure and functional potential of unculturable microorganisms. However, besides recent studies on lithic dryland habitats in Namibia and rain-affected Atacama soils (8, 13, 21), studies on microbial soil communities in deserts have primarily focused on analyzing total DNA, e.g. (7,11–13,22), lacking the ability to differentiate between sequences derived from living, potentially active communities and those originating from relic organisms (22). To address this limitation, a novel extraction method was developed. This method enables the separate recovery of intracellular DNA (iDNA) and extracellular DNA (eDNA) from the same sample, facilitating the analysis of DNA derived specifically from intact cells, including living and dormant microorganisms (23, 24). For this, cells and DNA are first washed out of the sediment using multiple rounds of specific buffer incubations and low-speed centrifugations and then filtered through a 0.22-µm filter to collect the cells. After this, the iDNA can be extracted from the filter using a phenol–chloroform protocol. This approach provides a significant improvement for microbial diversity studies of extreme environments as it effectively eliminates bias from DNA derived from dead cells, thereby ensuring more accurate and reliable results for understanding microbial life in extreme environments. Furthermore, it offers the opportunity to assess potentially active microorganisms in habitats with low biomass, where conventional RNA-based activity analysis reaches the detection limit due to low biomass.

The aim of this study was to explore microbial diversity in previously uncharacterized, deeper (>1 m) subsurface sediments of the hyperarid Atacama Desert and test whether the deep sediments in this region can represent a niche for specialized microbial communities. Therefore, we selected a sampling site located in a playa of the Yungay Valley, one of the driest places of the hyperarid core of the Atacama Desert (2). Playas are small and closed ephemeral wetlands that typically form in semiarid to arid regions and are characterized by accumulations of clay and evaporation features (25, 26). The sampling location differs from previous Atacama Desert study sites, as the vesicular layer, which is a common surface feature consisting of gypsum and anhydrite typically found close to the surface within the upper 50 cm, is buried underneath the playa sediments at a depth of approximately 2 m. We employed iDNA analysis (NGS and quantitative PCR, qPCR) and compared it to geochemical analysis (X-ray diffraction, XRD; ion chromatography (27)) to study the microbiology of the hyperarid subsurface down to a depth of 420 cm, including both playa sediments and alluvial fan deposits.

## Study site

### Profile description and mineralogy

**Fig. 1.**
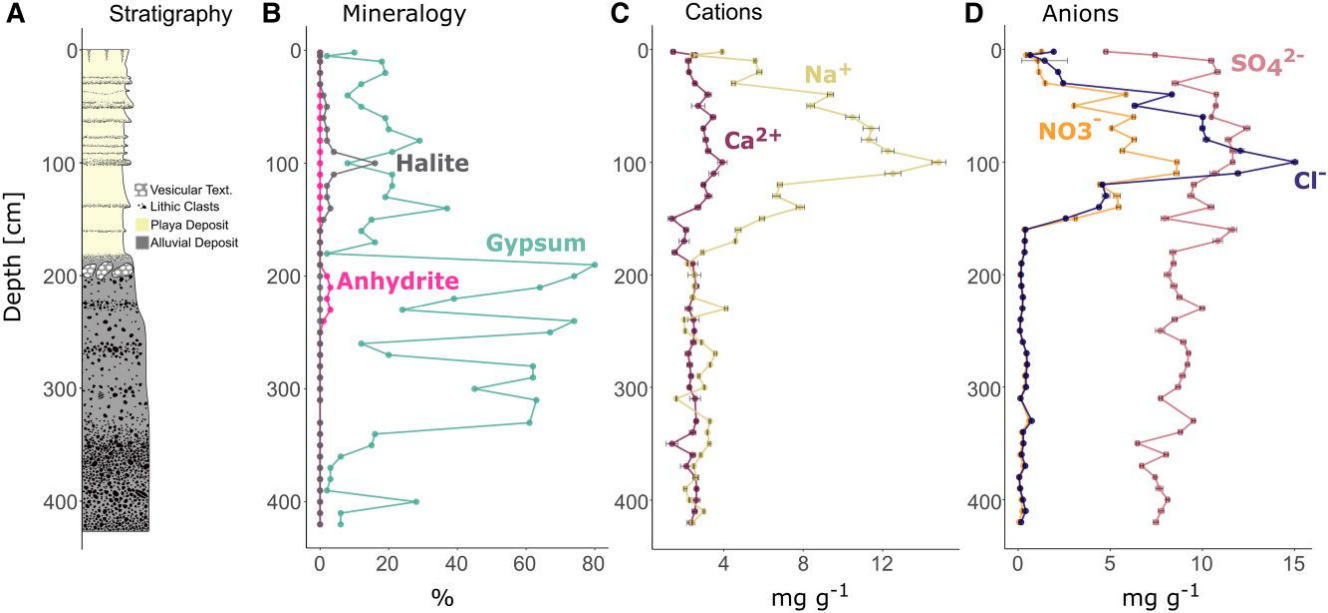
Stratigraphic column A) and geochemical attributes of the paleo profile plotted against depth according to Arens et al. (27). Mineralogy B) was measured semiquantitatively using XRD and is limited to the relevant salts in this study. Cation C) and anion D) concentrations per dry weight (dw) were acquired using ion chromatography. Error bars indicate the standard derivation of three technical replicates.

The exact same soil profile was previously investigated under a paleoclimatic aspect and consisted of two distinct zones based on grain size distribution (27). The upper half of the profile down to a depth of 184 cm consisted primarily of silty sediments with intermittent thin sand layers (Fig. 1A). Desiccation cracks, characteristic of fine playa sediments, were observed on the surface and within deeper layers down to 10–20 cm depth. Between 184 and 230 cm depth, the sediment transitioned to coarser textures, including sand and pebbles. Below 230 cm, the profile was consistently contained of pebble- to cobble-sized grains. During excavation, salt accumulations of gypsum (CaSO_4_ × 2H_2_O), anhydrite (CaSO_4_), and halite (NaCl) were encountered (Fig. 1B). The first major saline horizon composed of halite was found between 40 and 150 cm depth. Based on XRD data, halite amounts peaked at 100 cm depth accounting for 16% of the overall mineralogy. Gypsum was detected across the entire profile with a mean proportion of 16% in the upper part of the profile between 0 and 180 cm. Deeper sediments between 180 and 330 cm depth were characterized by degraded remnants of the sulfate-rich vesicular layer. This observation also correlates with higher proportions of gypsum in the XRD data showing average amounts of 53% and peaking at 190 cm depth with a value of 80%. Small amounts of anhydrite (1–3%) appear in association with gypsum between 200 and 240 cm depth. Below 330 cm depth, gypsum concentrations were decreasing to values below 10%.

### Geochemistry

Generally, sodium, calcium, sulfate, nitrate and chloride ions could be measured after leaching with MilliQ water (27). According to the ion concentrations, the profile can be divided into two main sections. In the upper third of the profile sodium, sulfate, nitrate, and chloride concentrations form a broad peak between 40 and 110 cm depth (Fig. 1C and D). Maximum concentrations for sodium (14.8 mg g^−1^ [dw]), nitrate (8.6 mg g^−1^ [dw]), and chloride (15.0 mg g^−1^ [dw]) were measured in sediments from around 100 cm depth and decreased between 150 and 200 cm depth (Fig. 1C and D). Moderate sodium concentrations (1.6–4.1 mg g^−1^ [dw]) defines the lower profile section from 150 to 420 cm depth, whereas calcium and nitrate concentrations in this section are low (<1 mg g^−1^ [dw]). Calcium and sulfate ions were constantly detected throughout the entire profile with concentrations around 2.5 mg g^−1^ (dw) for calcium and values between 4.8 mg g^−1^ (dw) and 12.4 mg g^−1^ (dw) for sulfate (Fig. 1C and D). Notably, 12.4 mg g^−1^ (dw) in our method corresponds to 2,000 mg l^−1^, which is close to the maximum solubility of gypsum (2,500 mg l^−1^ at 20 °C) explaining why the sulfate data are not following the trends of the relative amount of gypsum detected using XRD. We therefore, consider the XRD data to be more reliable for evaluating the presence of gypsum in the soil layers.

## Results

### Microbial composition and diversity

#### Data generation and sequencing statistics

Overall, 16S rRNA gene sequencing generated 10,632,339 reads resulting in the identification of 9,992 unique amplicon sequence variants (ASVs). Removal of low-quality, mitochondria, chloroplast, and contaminant sequences reduced the number of ASVs to 9,605 accounting for a loss of 3,933,284 reads. In addition, only 23 samples from 36 samples which were send to sequencing showed consistency between at least two replicates. From the remaining samples, e.g. the upper surface sample between 0 and 2 cm depth as well as all the samples from 100 to 190 cm depth, no reproducible, reliable sequencing data could be recovered. Therefore, these samples were removed from the dataset.

#### Microbial abundance and diversity

**Fig. 2.**
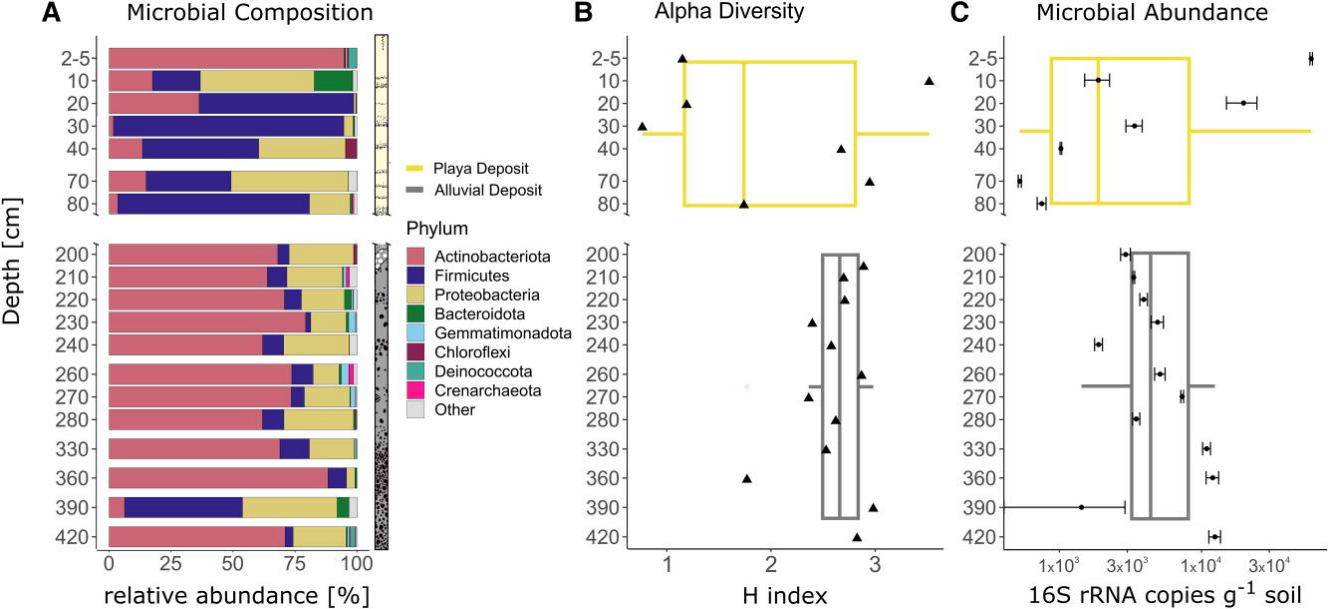
16S rRNA gene data showing the microbial composition A), diversity B), and abundance C) plotted against depth. Microbial composition is depicted at phylum level. Alpha diversity is shown as Shannon index values (*H*) and was calculated using a subsampled dataset to compensate for varying sequencing depth of the samples. Microbial abundance was measured using qPCR and was normalized to number of 16S rRNA gene copies g^−1^ soil. Note that the scale for the microbial abundance plot C) is logarithmic.

Gene copy numbers of iDNA were highly scattered across the upper 80 cm, ranging between 5.9 × 10^4^ copies g^−1^ soil found at 5 cm depth and 751 gene copies g^−1^ soil at 80 cm depth (Fig. 2C). Below 200 cm depth, gene copy numbers were relatively constant with an average of 4.4 × 10^3^ g^−1^ soil. Highest gene copy numbers in the lower profile section were observed for the samples collected at 330, 360, and 420 cm depth all exceeding 10^4^ gene copy numbers per gram soil, while the samples from the 240 and 390 cm horizon showed the lowest copy numbers with 1.8 × 10^3^ g^−1^ soil and 1.4 × 10^3^ g^−1^ soil, respectively.

Microbial diversity across the paleo profile samples was evaluated by assessing the number of observed ASVs (richness) and calculating the Shannon index. While microbial richness solely accounts for the number of different ASVs present, the Shannon index incorporates both the richness and the abundance of each ASV. Reported diversity indices were normalized to 24,755 sequences. Shannon values ranged from 0.8 to 3.5 with a median of 2.6. Observed richness ranged between 119 and 215 with a median of 164 observed ASVs (Fig. 2B). Diversity measurements suggest two distinct microbial communities across the paleo profile. Diversity metrics varied in the upper part of the profile between 0 and 80 cm depth, with values of *H* = 0.77 at 30 cm depth and *H* = 3.52 at 10 cm depth. Highest richness values in this profile section were observed at 20 cm depth with 180 observed ASVs, while at 40 cm depth the lowest richness of 133 ASVs was detected. Sediments from the lower profile section from 200 cm downwards were characterized by on average higher Shannon values, ranging from 1.8 to 3.0, being highest at 390 cm depth (*H* = 2.98). The number of ASVs ranged between 119 and 215 in samples from this section and were highest in samples from 215 cm depth.

**Fig. 3.**
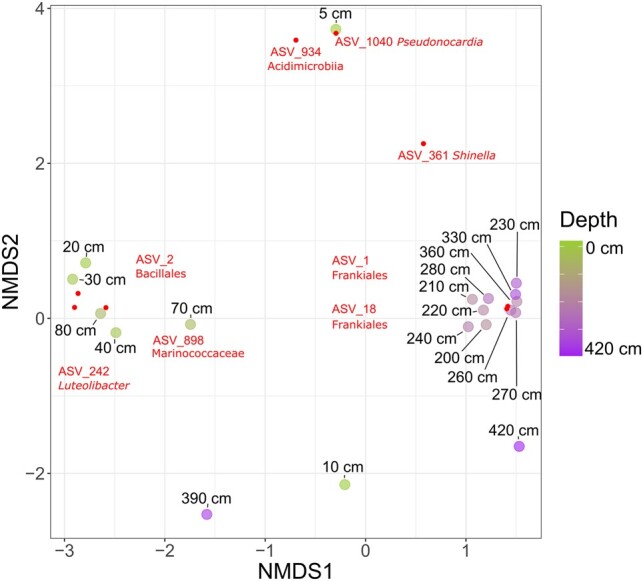
An NMDS displaying sample dissimilarity based on a Bray–Curtis dissimilarity matrix. The stress value of the NMDS plot is 0.12. Small dots indicate ASVs which have a significant (*P* < 0.01) influence on sample distribution in the ordination space. Each ASV's name and taxonomy are labeled in close association with the dots.

Beta diversity was assessed using Bray–Curtis dissimilarity between samples. A nonmetric multidimensional scaling plot (NMDS) based on the dissimilarity matrix revealed three distinct clusters, suggesting microbial communities to vary across the paleo profile (Fig. 3). Samples from the lower subsurface profile section (below 200 cm) clustered together in the right part of the ordination graph around NMDS1 = 1.5 and NMDS2 = 0, except for the communities at 390 and 420 cm depth. Samples in this cluster showed a stronger presence of ASV 1 and 18 (Tables S3 and S4), both classified to the bacterial order Frankiales, which's members are commonly inhabiting root nodules. The second cluster was more scattered and included samples from 20 to 80 cm depth. Three ASVs were characteristic for this cluster including ASV 2 (Bacilliales), ASV 242 (*Luteolibacter* sp.), and ASV 898 (Marinococcaceae). The surface sample from 2 to 5 cm depth was most distinct from every other sample, clustered separately and was especially defined by the presence of ASV 934 (Acidimicrobiia) and ASV 1040 (*Pseudonocardia* sp.). The communities from 10, 390, and 420 cm depth did not cluster with any of the before mentioned profile sections. While the deepest community from 420 cm depth was still in close spatial proximity of the lower subsurface cluster on the right side of the plot, the 10 and 390 cm communities were plotting far removed from all other community associations in the lower section of the NMDS plot.

#### Microbial community structure

Most of the sequences were assigned to bacterial taxa, while only 0.5% of the reads were assigned to Archaea. In general, microbial communities were dominated by three different bacterial phyla which account for over 90% of all sequences. The most abundant phylum was Actinobacteriota (51%) followed by Firmicutes (24%) and Proteobacteria (21%). The remaining identified phyla including Bacteroidota, Gemmatimonadota, Chloroflexi and Deinococcota accounted for <2% of the overall community. The most abundant archaeal phylum was Crenarchaeota with an overall relative abundance of 0.3%.

The uppermost sediments from 2 to 5 cm depth were dominated by the presence of Actinobacteriota (95%, Fig. 2A). Actinobacteriota were less abundant in sediments between 10 and 80 cm depth, ranging between 1.7% (30 cm) and 36% (20 cm) relative abundance. Microbial communities in this part of the profile were characterized by a strong presence of Firmicutes with relative abundances ranging from 47% (at 40 cm depth) to 93% (at 30 cm depth). Only the 70 cm sample was characterized by a lower relative abundance of Firmicutes at 34%. Microbial communities in sediments below 200 cm were again dominated by Actinobacteriota. Sequences classified to this phylum consistently appeared down to a depth of 420 cm with minimum abundances of 62% at 240 and 280 cm depth and a maximum abundance of 88% at 360 cm depth. Firmicutes abundance decreased significantly in sediments below 200 cm with relative abundances below 15%. However, Firmicutes were the most dominant taxon in sediments from the 390 cm deep sample, with a relative abundance of almost 50%. Proteobacteria distribution was more constant across the entire profile. Proteobacteria were especially enriched in sediments from the upper part of the profile with relative abundances of 46% at 10 cm depth, 35% at 40 cm depth, and 47% at 70 cm depth. In deeper sediments (below 200 cm), Proteobacteria abundance ranged between 10% (260 cm) and 38% (390 cm), but Proteobacteria were almost absent from 360 cm deep sediments.

To get a more detailed understanding of the microbial community and identify key organisms and their potential role in the unique Atacama Desert subsurface ecosystem, the most abundant and frequently occurring ASVs were evaluated more closely. ASV level characterization further emphasized the presence of two distinct communities existing in the upper sediments (down to 80 cm) and deeper sediments (below 200 cm).

**Fig. 4.**
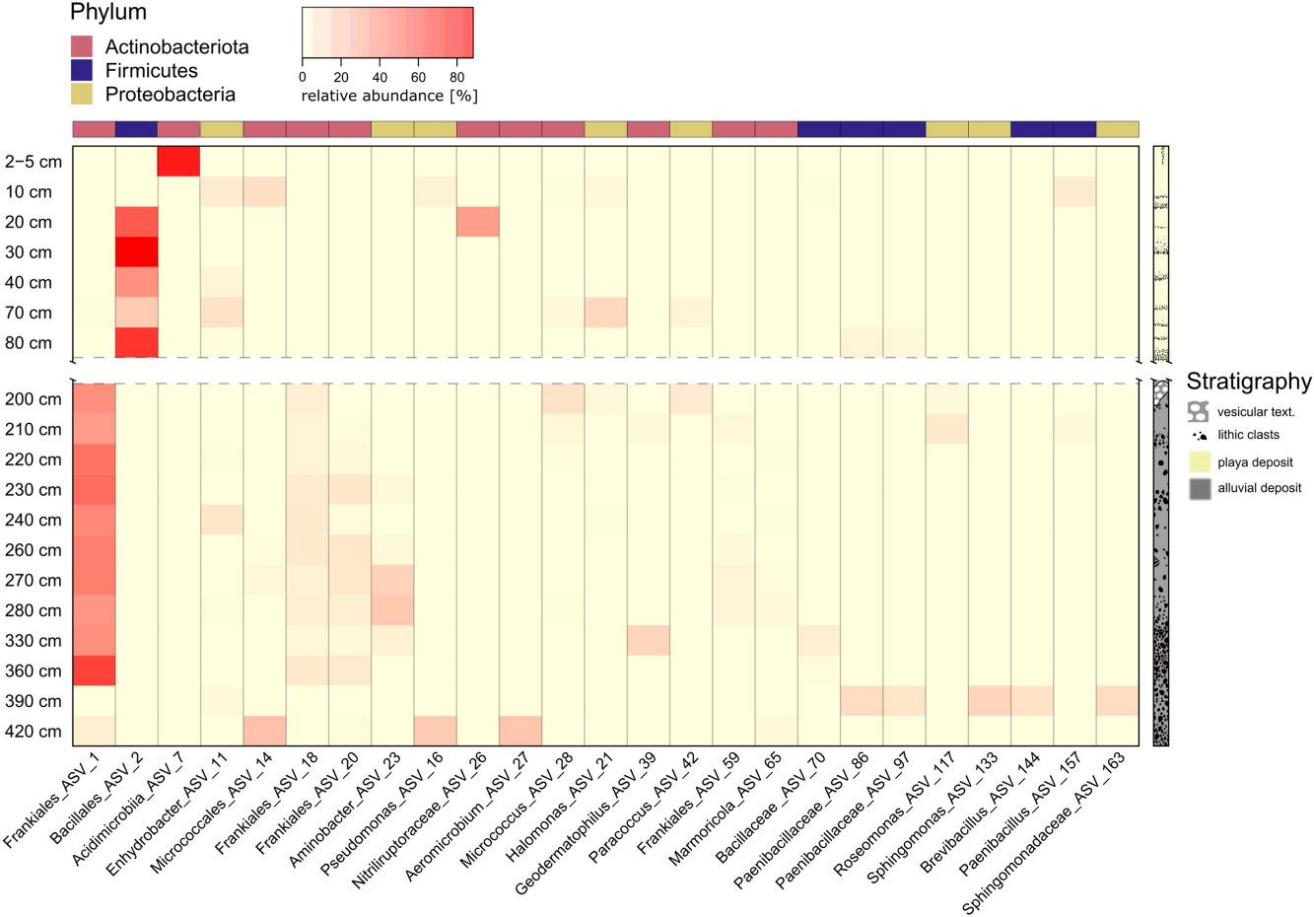
Heatmap showing the relative abundance of the dominant ASVs (mean abundance > 0.5%) and their highest assignable taxonomic rank. A schematic stratigraphic column shows sample association to specific profile zones and highlights the gap between 80 and 200 cm depth. For true to scale representation of the stratigraphy, see Fig. 1.

Actinobacteriota dominance in surface sediments (2–5 cm depth) could be attributed to the presence of ASV 7 (86% of all reads at this depth). ASV 7 was assigned to the Acidimicrobiia, but could not be classified below the class level (Fig. 4).

Firmicutes dominance in sediments taken from 10 to 80 cm depth was driven by ASV 2, which was assigned to the order Bacillales. ASV 2 was highly abundant (>50%) in this section of the profile, reaching up to 88% relative abundance in 30 cm deep sediments. The only notable appearance of Actinobacteriota in this profile section was observed at 20 cm depth, where ASV 26, assigned to the family Nitriliruptoraceae was determined to have a relative abundance of 33%. Proteobacteria communities in 40 and 70 cm deep sediments were especially enriched in two ASVs assigned to the genera *Enhydrobacter* (ASV 11; 4 and 11%) and *Halomonas* (ASV 21; 0.04 and 13%).

The dominance of Actinobacteriota in the subsurface sediments below 200 cm depth could be attributed to the presence of several ASVs (Fig. 4). The most abundant ASV (ASV 1) could be assigned to the order Frankiales, with abundances ranging from 34 to 65%. Other abundant Frankiales ASVs were ASV 18, ASV 20, ASV 59, and ASV 3. Most of them cooccurred with ASV 1. A similar pattern was observed for ASV 28 belonging to the order Micrococcales, albeit at lower relative abundances (0.03–10%). Only a small number of Actinobacteriota ASVs were observed in sediments from 390 cm depth. However, in the deepest profile sediments at 420 cm depth, they were abundant again, specifically with ASV 14 (21%, Micrococcales) and ASV 27 (20%, *Aeromicrobium* sp.).

ASVs belonging to the Proteobacteria were characterized by ASV 23, assigned to the genus *Aminobacter*. This ASV was especially enriched in sediments from 270 and 280 cm depth, with relative abundances of 15 and 19%, respectively. Another abundant Proteobacteria ASV belonging to the genus *Pseudomonas* (ASV 16, 18% relative abundance) was detected in the deepest sample. Other, less abundant Proteobacteria ASVs in the deeper part of the profile, were ASV 11 affiliated to the genus *Enhydrobacter* (reaching 8% at 240 cm depth) and ASV 42 affiliated to the genus *Paracoccus* (reaching 7% at 200 cm depth).

Firmicutes ASVs were detected infrequently in the deeper sediments. Two Firmicutes ASVs assigned to the genus *Brevibacillus* (ASV 144) and to the family Paenibacillaceae (ASV 97) were identified at abundances of 15 and 12% in the 390 cm sample, respectively. Across the remaining horizons only one Firmicutes ASV was detected at relative abundances above 1%, with ASV 70 assigned to the family Bacillaceae at 330 and 360 cm depth.

## Discussion

The extraction and sequencing of iDNA from sediment samples collected from a soil profile in the Atacama Desert allowed the assessment of potentially active communities in an exceptionally oligotrophic habitat where common activity proxies such as metatranscriptomics often fail due to the low abundance of extractable RNA and the fast decay of RNA (28, 29). This investigation revealed potentially viable microbial communities to inhabit the hyperarid soils down to a depth of at least 420 cm. To our knowledge, this represents the deepest microbial survey and discovery of microbial life in Atacama soils to this day with previous studies mostly covering only shallow subsurface soils to a depth of 80 cm, e.g. Warren-Rhodes et al. (11) and Schulze-Makuch et al. (8), with only two exceptions reaching down to 340 and 520 cm depth (19, 20). Considering the lack of input of nutrients from atmospheric sources in the deeper subsurface of deserts, communities described in this study could represent the upper extent of a deep biosphere underneath hyperarid desert soils.

### Depth-related diversity trends

Diversity in Atacama soils is reported to decrease with depth suggesting harsher conditions in the subsurface of the desert (11). In the upper meter of the soil profile, a similar trend was observed as gene copy numbers decreased from 5 to 80 cm depth. Furthermore, it was observed that the diversity of microbial communities in this specific profile section tended to be lower compared to previously reported values (7, 11). The diversity values recorded in this study, with a median Shannon index (*H*) of 1.8 and a minimum value of 0.8 at a depth of 30 cm, are lower than the values of 2.6 observed at the study site Kevin Garden and 2.1–3.5 in the surface playa soils as reported by Crits-Christoph et al. (7) and Warren-Rhodes et al. (11), respectively. As indicated by the measured chloride, nitrate, and sodium concentrations, in addition with an increase of halite in the mineralogy, saline conditions are increasing with depth within the upper meter of the paleo profile. Therefore, consistent to previous observations (11), the overall low diversity and decrease in biomass is likely connected to the higher osmotic stress on the microorganisms. No reliable microbial community data could be collected for sediments from around 100 cm depth, where ion and mineralogy data suggest salinity to be highest. Despite microorganisms are known to withstand very high salt concentrations inhabiting salt nodules in surface soils, e.g. de los Ríos et al. (12) or even benefit from deliquescence processes in the subsurface (20), osmotic regulation requires substantial energy supply which is achieved through energetically beneficial metabolisms like photosynthesis, aerobic respiration, and denitrification (30). We propose that under the absence of sunlight and limited organic matter halophilic life is limited and thus living biomass is too low for sufficient iDNA yield in subsurface layers where salinity is peaking. However, the gap in iDNA recovery persists beyond the extent of the highly saline layers to the bottom of the playa deposits down to a depth of 190 cm. An explanation for this could be the isolation from water and nutrient input in sediments below the chloride- and nitrate-rich horizons as these salts in desert soils often mark the maximum infiltration depth of water during rare rain events (3). It is not clear whether such an infiltration process could also act in playa sediments, since clay rich deposits are considered to seal off water. Playas are known to serve as an important landform for groundwater-recharge where infiltration rates can be high due to waterflow along desiccation cracks (31). Such an infiltration could indeed be responsible for the deposition of nitrate and chloride salts around 100 cm depth in the playa sediments. However, the climate record of the playa sediments also shows that the onset of higher nitrate and chloride concentrations correlates with higher fluvial activity in the basin, implying the salts to be transported and deposited from the surrounding slopes (27). Independent from the origin of the salt accumulation, the deeper playa layers are more isolated from nutrient and water input than the upper 80 cm which highly limits the colonization and persistence of microbial communities in the layers between 150 and 200 cm depth. Microbial diversity below 200 cm depth was found to be stable (*H* index between 1.8 ando 3.0), showing no depth-related trend. This observation suggests a mostly substrate-dependent community which probably colonized the alluvial deposits prior to its burial underneath the playa sediments. Overall, this indicates a transition zone at around 2 m depth, which is impacted by the precipitation of salts, reaching a fairly isolated deeper subsurface that is unaffected by surface processes.

### Specialized communities in playa sediments (0–80 cm)

The community composition in the upper playa sediments of the soil profile exhibits significant fluctuations. Reliable sequencing data could not be obtained from little amounts of DNA recovered from the surface (0–2 cm depth) of the profile, likely due to the low biomass. This aligns with previous observations conducted in the Yungay area (10, 32). The microorganisms at the soil–atmosphere interface in the desert faces stress from diurnal temperature fluctuations, desiccation, and intense UV radiation (1). To escape these harsh conditions endolithic bacteria often colonize rocks or salt crystals, e.g. Lacap-Bugler et al. (15), de los Ríos et al. (12), and Genderjahn et al. (21), providing niches which allow microbes to be permanently active (33). We hypothesize that the absence of larger salt crystals or minerals within the fine particles of the playa deposits prevent potential endolithic life as there is no shelter from the extreme environmental conditions, e.g. the colonization by cyanobacteria on/in surface sediments, e.g. (12, 15). Furthermore, endolithic life relies on moisture supply which has been reported to be insufficient in surface soils of Yungay (17). Based on these observations, we conclude that the lack of reliable sequencing data in the upper 2 cm of the soil profile are due to limited microbial colonization caused by the harsh surface conditions and the lack of potential microniches to provide refuge from these conditions.

The 2–5 cm sample had a high abundance of taxa belonging to Actinobacteriota, a phylum known to dominate the soils of the Atacama Desert (7, 8, 10). However, this particular horizon exhibits a remarkable presence of an uncultured bacterium belonging to the class Acidimicrobiia, which constitutes 86% of the entire sample. While this ASV was almost only observed in the 2–5 cm horizon of the paleo profile, members of the same class have regularly been encountered in previous studies of the Atacama Desert and are considered part of the native core community which is present at different sites and soil depths (7, 8, 11). The identified ASV 7 is closely affiliated with GenBank entries of another uncultured Acidimicrobiia bacterium (e.g. 99.21%, accession: OW750262.1) extracted from desert soil. Moreover, members of the Acidimicrobia are native to deserts around the world as the isolation of a novel organism from soils in the Chinese Gurbantunggut desert led to the proposal of the Acidimicrobiia species *Desertimonas flava* (34). The ASV 7 identified in the 2–5 cm horizon likely represents a highly specialized and undescribed organism that is well adapted to desiccation. This bacterium thrives in the surface layers unaffected by UV radiation, where salt concentrations remain relatively low due to occasional dissolution during rare rain events. The absence of this ASV in the other layers of the upper sediments is likely attributed to increasing salt contents of the sediments below 5 cm depth. Similar to other Acidimicrobiia native to desert environments, the here identified ASV may have the genetic potential for hydrogenotrophic carbon fixation (35). The flexibility to switch to a chemolithotrophic metabolism could offer this ASV the opportunity to persist and compete over long times with limited external nutrient supply.

From 20 to 80 cm depth, where the salt concentrations start to increase, the microbial community is defined by a dominance of Firmicutes. This dominance is almost entirely facilitated by ASV 2 closely related to *Scopulibacillus darangshiensis* (98.02%; accession: FN646630.1), *Salinibacillus xinjiangensis* (96.84%; accession: NR_125634.1), and *Alteribacillus bidgolensis* (96.84%, accession: NR_109122.1), which are halophilic and strictly aerobic organisms isolated from hypersaline lakes (36, 37). This is consistent with the accumulation of chloride and nitrate salts in this zone and observations of viable, halophilic Firmicutes taxa in the hyperarid core of the Atacama (8, 20). Salinity between 20 and 80 cm depth is, therefore, not only influencing diversity but also defining the community composition of this profile section.

The fine-grained playa sediments are usually deposited during times of temporal lake formations in the basin. Such temporary wetting phases have most likely a major impact on the input of microbial communities to the playa sediments. Many Firmicutes are endospore-forming organisms; therefore, it is possible that the Firmicutes communities inhabiting the saline layers of the paleo profile are dormant under the current dry conditions and become active during the short flooding events of the basin. In our study, iDNA was extracted using a phenol–chloroform-based cetyltrimethylammonium bromide (CTAB) protocol with bead beating which could lyse endospores. However, Schulze-Makuch et al. (8) could prove decreasing influence of sporulation biomarkers with increasing aridity, indicating that the Firmicutes taxa in the upper meter of the studied profile could be potentially vegetative.

Overall, the microbial community of the first meter of the paleo profile is highly adapted, with halophilic Firmicutes being abundant across most of the samples and suggesting depth-related salt concentrations to be the main factor shaping microbial community and diversity in playa sediments.

### A gypsum-dependent deep subsurface niche

One of the most notable findings from this study is that microbial cells were recovered from sediments below 200 cm depth where the playa deposits transition into alluvial deposits. Contrary to assumptions which were based on previous patterns observed in other subsurface environments (11), microbial diversity and abundance did not decrease in deeper sediments. The potentially viable communities between 200 and 420 cm depth showed a consistent Shannon *H* diversity of around 2.7 which is comparable to values from previous studies on Atacama surface soils (7, 11). Although gypsum is present across the entire profile, gypsum as a major part of the vesicular layer was only observed within the coarser sediments of the alluvial fan deposits. Porous gypsum has been observed to be inhabited by endolithic microorganisms that utilize the pore spaces as an ecological niche in various environments, e.g. Hughes and Lawley (38) and Wierzchos et al. (17). These endolithic communities near the surface often consist of phototrophs, which form a consortium alongside heterotrophic bacteria closely related to taxa found in the surrounding soils (12, 15). The structure of gypsum crystals is known to provide protection against harmful UV radiation, while also being translucent and retaining moisture within the pore space (17, 18). Cyanobacteria are even reported to actively dissolve gypsum crystals to recover the water incorporated into the mineral structure (39). However, the gypsum crystals subsurface alluvial deposits of the paleo profile were not colonized by cyanobacteria due to missing light, but creating a niche that is inhabited by specialized microorganisms. Between 200 and 240 cm depth, gypsum was accompanied by small fractions of anhydrite. This subsurface layer contains the remnants of the vesicular layer which has been investigated in surface soils of the surrounding slopes and shown to be a capable to maintain microbial activity over a longer period compared to the surface samples (8). Biogenic-induced dissolution of gypsum by bacteria has been speculated to cause partial transformation of initial gypsum to anhydrite due to the loss of water and the reprecipitation of CaSO_4_ (39). However, experiments showed that the direct dehydration of gypsum to anhydrite requires high temperatures generally above 85 °C (40, 41) and it is unclear if microbes can facilitate this transformation. Recently, the formation of anhydrite in the Atacama has been attributed to secondary dissolution of gypsum and reprecipitation of anhydrite which could be thermodynamically favored by high concentrations of nitrate and halite salts (42, 43). In this study, the occurrence of anhydrite is not accompanied by higher concentrations of nitrate or halite but with the presence of an Actinobacteria-dominated microbial community. Additionally, the lower sections of the alluvial fan deposits, below a depth of 340 cm where the gypsum content decreased, gene copy numbers reached a minimum value at 390 cm depth. This together with previous evidence of gypsum supporting microbial life in the Atacama (8, 17, 18, 39) further suggests gypsum to play a crucial role for microbial biodiversity by possibly providing water or increasing water retention in hyperarid soils.

Another major difference between the upper playa deposits and the lower alluvial fan deposits is their deposition age. The fine-grained playa sediments are relatively young, with sedimentation starting approximately 19,000 years ago (27). In contrast, the alluvial deposits represent a much older depositional record, dating back up to 3.8 million years at a depth of 420 cm (27). The age of the alluvial fan deposits, coupled with the persistence of the habitat, may contribute to the higher diversity and community stability observed, resulting in the selection of taxa that are adapted to the desert soil conditions. Long-lasting environments not only accumulate active microbial members but also allochthonous DNA (44, 45), which could be especially true for low activity settings where DNA is not as quickly consumed as in other environments. Furthermore, certain materials like clay or salts bind eDNA, making it unavailable for consumption and thus additionally contribute to the accumulation of eDNA (46, 47). In our study, we were able to separate this potential bias by specifically analyzing the iDNA pool which enables us to directly look at the members of the potentially viable subsurface community.

The community composition of the alluvial fan deposits in this study is comparable to previous studies conducted on surface sediments in the Atacama Desert, where Actinobacteriota have been identified as the dominant phylum (7, 8, 10, 48). Within the Actinobacteriota, the assigned ASVs belonging to the order Frankiales are affiliated with the species *Geodermatophilus pulveris* (ASV 18; 97.23% similarity) and *Modestobacter caceresii* (ASV 1; 97.23%), both of which have been previously found in desert habitats (49–51). Notably, *M. caceresii*, isolated from hyperarid Atacama soils, demonstrates remarkable genetic capacities to withstand the diverse challenges in this extreme desert environment. It exhibits resistance to temperature fluctuations, osmotic stress, UV radiation, and low nutrient conditions, while also possessing the potential for a chemolithotrophic metabolism, utilizing CO as the sole carbon and energy source (50). Other Actinobacteriota identified in the lower subsurface, including ASVs belonging to the order Micrococcales (ASV 14 and 28), were also identified in previous Atacama Desert studies (8). This suggests that this particular community colonized the soil prior to its burial by the playa deposits 19,000 years ago and that it was flexible enough to persist under the extremely nutrient isolated conditions in the deeper subsurface of the desert. Hydrogenotrophic carbon fixation is known to be an important process in hyperarid soils dominated by Actinobacteriota classes which possess the genes to fix CO_2_ by oxidizing atmospheric hydrogen (35). Symbiotic and free-living Frankia, a common genus within the order Frankiales dominating the lower subsurface deposits in the paleo profile, are typically able to fix atmospheric nitrogen while also having the metabolic potential to oxidize hydrogen (52–54). The close affiliation of the Frankiales ASVs to potentially chemolithoautotrophic organisms (*M. caceresii* (50)) and the absence of recent phototrophically produced organic carbon highlights the importance of mixotrophy or specifically chemolithoautotrophy in subsurface layers below 2 m depth. The discovery of potentially active chemolithoautotrophic microorganisms in previously unknown subsurface niches changes our understanding of microbial life in desert soils. This finding indicates that the contribution of chemosynthetically produced carbon in deserts may have been significantly underestimated thus far.

### Active or dormant community?

Cell viability can be roughly defined by (i) the presence of functional nucleic acids, (ii) cellular activity, and (iii) membrane integrity (55). Our work demonstrates how iDNA analysis can help to better understand present ecosystems in extreme settings, including nutrient limitation, osmotic stress, and drought, which often lead to low biomass and thus, make RNA-based methods very challenging or even impossible. However, in contrast to metatranscriptomics or ATP analysis, iDNA cannot distinguish between active or dormant organisms as it is only referring to physiologically intact cells. In this study, we used the term “living communities” to describe the state of the cell assemblages assuming the fraction of dead cells with intact membranes to be negligible, especially considering the oligotrophic nature of the hyperarid sediments, where the decomposition of microbial cell walls as a nutrient source is expected to be more pronounced due to reduced water activity (56). Furthermore, a small overlap of ASVs between iDNA and eDNA in hyperarid soils of the Atacama Desert indicated very low cell turnover rates (8). We assume that low turnover rates could additionally mitigate the risk of overestimating the living community by minimizing the likelihood of extracting intact but deceased cells, since cell death is likely to appear more infrequently in comparison to more metabolically active habitats. Furthermore, spore formation is reported to be a minor factor in hyperarid Atacama soils, even when typical endospore-forming Firmicutes taxa were encountered (8). Therefore, microbial communities’ patterns in our study, such as the dominance of Firmicutes in playa sediments, do not necessarily imply a dormant community but rather may reflect snapshots of slow yet dynamic community processes leading to substrate-dependent shifts in community composition.

## Conclusions

This study expands our knowledge about microbial biodiversity of desert environments through the discovery of a previously undiscovered subsurface niche hosting a viable and potentially active community. By reaching down to a sampling depth of 420 cm covering playa sediments and underlying alluvial fan deposits, different community dynamics in regard to depth and soil type were observed. The upper 2 m of the profile are characterized by depth-dependent communities, as diversity and habitability decrease with depth due to increasing salinity and water scarcity, findings that correspond to previous observations in Atacama Desert soils (11). However, unlike previous investigation, we show that microbial communities reappear in the deeper alluvial fan deposits which could be connected to the presence of vesicular gypsum potentially offering an alternative water source through dissolution to anhydrite. This subsurface community is more diverse than the specialized surface communities found in the playa sediments and is likely completely isolated from the surface. Even though gypsum may not be ubiquitous in the subsurface of all deserts, the presence of this subsurface niche could indicate that the global biodiversity of deserts was underestimated so far and that under given circumstances a subsurface community can persist in the deeper layers of the driest places on Earth. Finally, this study also holds important implications for the search of extremophilic life beyond Earth. The exploration of gypsum-associated subsurface environments in the Atacama Desert has direct relevance to astrobiology, since gypsum deposits on Mars (57) are, as this study further underlines, not only evidence of past liquid water (58, 59) but could also possibly serve as a source of water for present microbial life. Thus, the data from this study is helping us to understand if and how life may exist in similar environments on other planets or moons across our solar system.

## Materials and methods

### Sampling

**Fig. 5.**
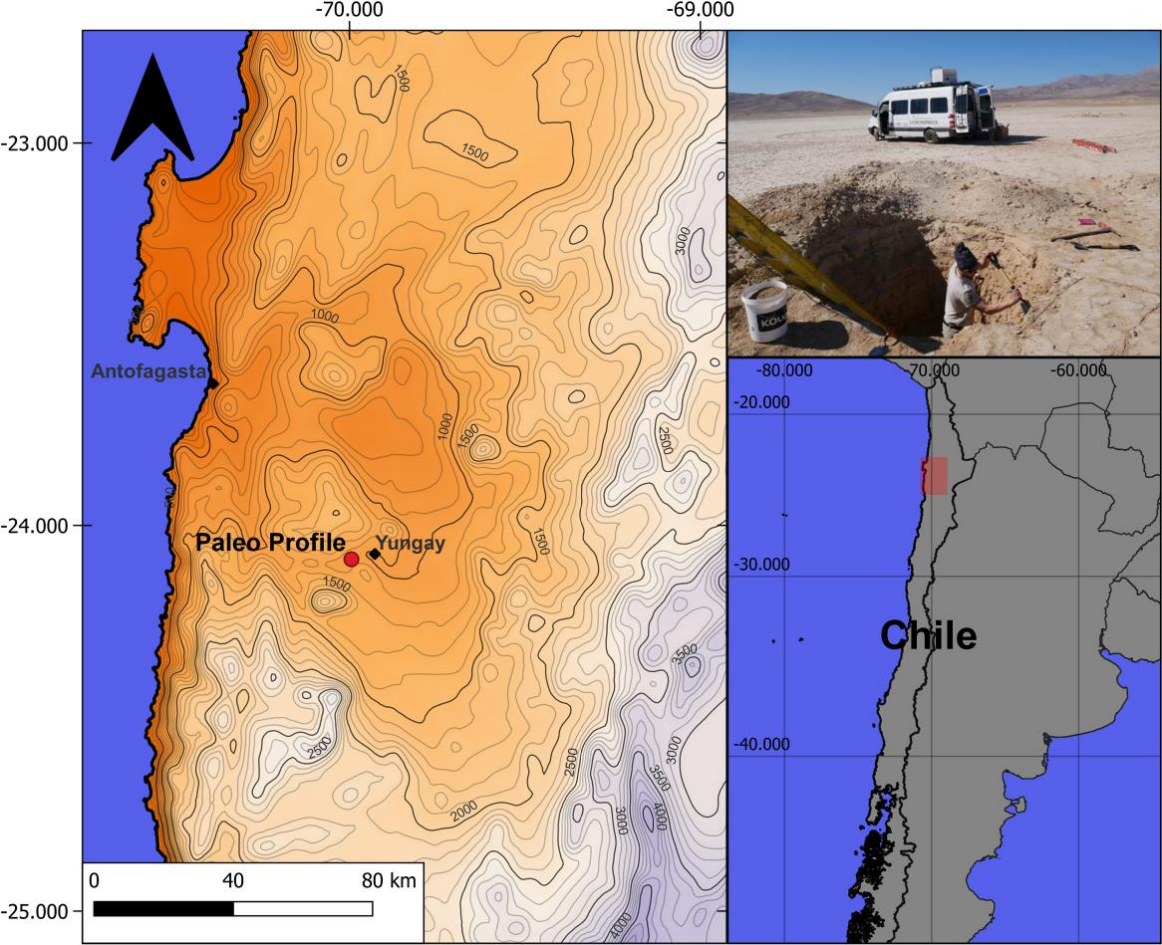
Map showing the location of the study site in the hyperarid core of the Atacama Desert.

The sampling site is a playa/clay pan situated in a closed basin located in the Yungay Valley of the Atacama Desert in northern Chile, approximately 60 km southeast of Antofagasta (24°05′41.4″S 70°01′42.7″W; Fig. 5). Sampling of deep soil profiles while providing sterile sampling conditions (e.g. avoiding the use of heavy drilling instruments) in this region is extremely challenging due to the highly consolidated nature of the desert ground. Therefore, the sampling was conducted over the time span of one month during March and April of 2018 and included the excavation of a 1.5- to 2-m deep, preexisting soil pit to a depth of 420 cm. Three pristine samples covering a lateral extend of 1 m for each layer were taken progressively while digging downwards excavating 50 cm laterally into the profile to reduce the influence of atmospheric gases diffusing into the soil layers.

The initial 5 cm of the soil profile were sampled in two segments: 0–2 and 2–5 cm in depth. Subsequently, samples were collected in 10 cm intervals. For the samples designated for biological analysis, a modified sampling interval of every 30 cm was implemented between 300 and 420 cm, resulting in a total of 36 samples. Each sample was carefully collected by sampling multiple spots across a meter wide horizontal layer, which were than pooled and homogenized to accurately represent the entirety of the excavated pit’s area. Sampling was performed using a flamed sterilized spoon transferring the sediment into sterilized 125 mL containers. Sampling containers were stored at ambient temperatures after sample collection and at 4°C during transport to Germany. Upon arrival in Potsdam, the samples were transferred to −20°C for long-term storage. In addition, a total of 44 samples were collected for geochemical analysis using Nasco Whirl-Pak bags and stored at room temperature until downstream analysis.

### DNA extraction

#### Separation of iDNA and eDNA

To separate iDNA and eDNA from sediment samples at different depths, a modified version of the protocol developed by Alawi et al. (23) and Medina et al. (24) was used. Three replicates per sample each containing 3 g of sediment were mixed with 6 mL of 120 mM sodium phosphate (NaP) buffer pH 8.0 (400 mL of 120 mM Na_2_HPO_4_ + 29.2 mL of 120 mM NaH_2_PO_4_, sterile filtered over a 0.2-µm PES filter and autoclaved) and incubated on an orbital shaker at 4°C for 10 min. The slurry was then centrifuged at 500*×g* and 4°C for 10 min to separate the sediment from the solution. This process was repeated 3 times by resuspending the resulting pellet in 3 mL of NaP buffer to maximize DNA and cells yield.

The separation of the two DNA fractions was achieved by filtering the collected supernatant through a 0.22-µm Sterivex filter (preflushed with NaP buffer) using a sterile 20-mL syringe, allowing intact cells (iDNA) to be captured on the filter membrane, while the eDNA remained in the buffer. To ensure complete transfer of the eDNA fraction, the filter was flushed with NaP buffer afterwards. All steps were performed in a sterile bench. The filter containing the iDNA was stored at −20 °C until DNA extraction.

#### Extraction of iDNA

For the extraction of iDNA, the frozen Sterivex filters were subjected to a CTAB–polyvinylpyrrolidone DNA extraction method based on Nercessian et al. (60). The filter membrane was cut and transferred to a sterile 2-mL screwcap tube. To lyse the cells, a mixture of zirconia and glass beads, 600 µL CTAB buffer, 60 µL 10% SDS, and 600 µL phenol–chloroform–isoamyl alcohol (25:24:1) were added to the tube, then vortexed for 45 s at 6 ms^−1^ using a bead beater. After bead beating, the tubes were centrifuged, and the upper aqueous phase containing the DNA was transferred to a new tube. This step was repeated with 600 µL chloroform–isoamyl alcohol (24:1) to remove any residual phenol. The DNA was precipitated with two volumes of PEG 6000/NaCl and 1 µL linear polyacrylamide, centrifuged, washed with 70% ethanol, air-dried, and finally dissolved in PCR-grade water. The extracted DNA was stored at −20 °C for further processing.

#### Library generation and NGS

In this study, the V4 region of the 16S ribosomal RNA gene was targeted using the universal primer pair F′515 (5′-GTGYCAGCMGCCGCGGTAA-3′; modified by Parada et al. (61)) and R′806 (5′-GGACTACNVGGGTWTCTAAT-3′; modified by Apprill et al. (62)) which is suitable for the amplification of both archaea and bacteria. PCR with barcoded primers was done using the following program in a Bio-Rad T100 Thermal Cycler: a first denaturation step at 95°C for 5 min followed by 35 cycles at 95°C for 30 s, annealing at 55°C for 30 s, and extension at 72°C for 1 min. The PCR products were purified using the Agencourt AMPure XP kit (Beckman Coulter Life Science, Krefeld, Germany), pooled and send for sequencing. Illumina paired-end sequencing was performed at Eurofins Genomics (Ebersberg, Germany) on an Illumina HiSeq machine with MiSeq V3 chemistry (2 × 300 bp paired-end reads).

#### Data processing

The sequencing library was demultiplexed using cutadapt v3.5 (63) using the following parameters: -e 0.2 -q 15,15 -m 150 --discard-untrimmed. The ASVs were generated using trimmed reads and the DADA2 package v1.20 (64) with R v4.1 using the pooled approach with the following parameters: truncLen = c(240,200), maxN = 0, rm.phix = TRUE, minLen = 200. Taxonomic assignment was done using DADA2 and the SILVA database v138 (65). Subsequently, ASVs representing chloroplasts, mitochondria, and singletons were removed.

Contamination was traced by running negative controls during DNA extraction as well as library preparation and including them in the sequencing library. Contaminating sequences, which showed comparable or greater abundance in at least one of the negative controls, were carefully removed from the dataset. In addition, extraction replicates were checked for consistency to eliminate contaminated or low sequencing read count data points. Samples for which two or more replicates had to be removed were not included in downstream analyses. Diversity analysis (alpha and beta diversity) was done on a subsampled dataset to avoid effects of different sample sizes. All samples were subsampled to a read count of 24,755 using R's vegan package (66) which corresponds to the sample with the lowest sequencing depth in this library. Data visualization on phylum and ASV level was done after the nonrarefied read counts were transformed into relative abundances. All plots were generated using the R packages phyloseq (67), vegan (66), and ggplot2 (68).

#### Quantitative PCR

The qPCR analysis was conducted using a CFX Connect Real-Time PCR detection system (Bio-Rad). A 20-µL master mix was prepared, consisting of 10 µL of KAPPA Hifi SYBR Mix 1 × (Qiagen), 0.4 µL each of 10 µM primers 341 F (5′-CCTACGGGAGGCAGCAG-3′), and 534 R (5′-ATTACCGCGGCTGCTGG-3′) (69), 5.2 µL of PCR-grade H_2_O, and 4 µL of DNA template. The qPCR cycling parameters were set as follows: an initial denaturation step at 95°C for 3 min, followed by 40 cycles of denaturation at 95°C for 3 s, annealing at 60°C for 20 s, elongation at 72°C for 30 s, and plate reading at 80°C for 3 s. All samples were analyzed in triplicate for technical replicates. To establish a standard curve and determine efficiency (>90 to <110%), a known concentration (2.5 × 10^8^ gene copies) of a 16S rRNA gene PCR fragment from *Bacillus subtilis* was used, with serial dilutions ranging from 10^1^ to 10^7^ gene copies. The Bio-Rad CFX software was employed for efficiency calculation. Extraction negative controls and qPCR nontemplate controls were included in the analysis. Additionally, a melting curve analysis was performed at the end of each run to detect any nonspecific DNA amplification (70).

## Supplementary Material

pgae123_Supplementary_Data

## Data Availability

The raw 16S rRNA amplicon data are available in the European Nucleotide Archive under the accession number PRJEB39249 or https://www.ebi.ac.uk/ena/browser/view/PRJEB39249. All other displayed data, including curated ASV tables, can be viewed in the Supplementary material. For more information on the geochemical/sedimentological aspect of the paleo profile, please view Arens et al. (27).
