# Supplementary material for: Persistent microbial communities in hyperarid subsurface habitats of the Atacama Desert: Insights from intracellular DNA analysis
Source: PNAS Nexus. 2024 Apr 23;3(4):pgae123. doi: 10.1093/pnasnexus/pgae123 (PMC11037274; doi:10.1093/pnasnexus/pgae123)
Supplement: pgae123_Supplementary_Data [file pgae123_supplementary_data.docx]

**Supplementary Information**

**Table 1** Showing the relative abundance (%) of the 8 most abundant phyla across the paleo profile.


**Table 2** Showing the most abundant ASV’s (average abundance of > 0.5 %) across the paleo profile.

**Table 3** Showing the taxonomic assignment of the ASV’s that significantly (p < 0.01) to the ordination of the samples in Fig. 3.

**Table 4** Showing the relative abundance across samples for each ASV that contribute significantly to the ordination of samples in Fig. 3.

**Table 5** Showing the assigned SILVA taxonomy of the most abundant ASV’s from table 2.

**Table 6** Showing the sequencing depth, microbial abundance and alpha diversity across the paleo profile.

**Table 7** Showing the geochemical data across the paleo profile including water soluble ions (per dry weight) and mineralogy measured via X-ray diffraction from Arens et al. (2023).
